# Mapping *Escherichia coli* in Women with Simple Urinary Tract Infections: Phenotypic ESBL/AmpC Screening and Whole-Genome Insights from Oman

**DOI:** 10.3390/antibiotics15020124

**Published:** 2026-01-27

**Authors:** Aisha Al-Mufarji, Meher Rizvi, Nawal Al-Kindi, Nada Al-Tamtami, Zaaima Al-Jabri

**Affiliations:** 1 Department of Microbiology and Immunology, Sultan Qaboos University, Muscat 123, Oman; 2 Directorate of Infection Prevention and Occupational Safety, Khoula Hospital, Ministry of Health, Muscat 112, Oman; 3 Department of Laboratory Medicine, AL-Masarrah Hospital, Ministry of Health, Muscat 100, Oman

**Keywords:** simple urinary tract infection (sUTI), multidrug-resistant (MDR), *Escherichia coli*, extended-spectrum β-lactamase (ESBL), antimicrobial susceptibility, carbapenem-sparing agents, whole-genome sequencing (WGS), antimicrobial resistance, antimicrobial stewardship

## Abstract

**Background/Objectives:** Simple urinary tract infections (sUTIs) are common in women and increasingly affected by multidrug-resistant (MDR) *Escherichia coli*. Extended-spectrum β-lactamase (ESBL) and AmpC producers restrict oral treatment options and promote carbapenem use. This study aimed to (i) describe the etiology and antimicrobial susceptibility of sUTIs in women of reproductive age in Oman, (ii) determine the prevalence of ESBL/AmpC-producing *E. coli*, (iii) evaluate nitroxoline, fosfomycin, mecillinam, and temocillin against ESBL and non-ESBL *E. coli*, and (iv) characterize circulating clones and resistance/virulence determinants using whole-genome sequencing (WGS). **Methods:** In this multicentric study (September 2022–August 2023), 795 uropathogens from 762 women (15–50 years) with sUTI were collected from four Omani hospitals. Identification and susceptibility testing of *E. coli* (n = 489) and *Klebsiella pneumoniae* (n = 140) using BD Phoenix and MALDI-TOF MS was performed (CLSI 2022). Thirty ESBL-producing and 82 non-ESBL *E. coli* underwent phenotypic ESBL/AmpC testing and evaluation of mecillinam, temocillin, nitroxoline, and fosfomycin. WGS was performed on 26 isolates (23 ESBL, 3 wild type) and analyzed for MLST, and SNP phylogeny using ResFinder, CARD, PlasmidFinder, VirulenceFinder. Statistical significance was set at *p* < 0.05. **Results:** *E. coli* (62%) and *K. pneumoniae* (18%) were the predominant pathogens. *E. coli* showed high susceptibility to nitrofurantoin (~97%), carbapenems, aminoglycosides, and piperacillin–tazobactam, but reduced susceptibility to cephalosporins, fluoroquinolones, cotrimoxazole, and ampicillin. ESBL prevalence ranged from 38–51%; AmpC producers were rare (4.6%). Mecillinam, nitroxoline, and fosfomycin exhibited 100% activity against both ESBL and non-ESBL isolates; temocillin showed 89.3% activity in ESBL strains. WGS identified 15 sequence types dominated by ST-131, ST-1193, ST-73, and ST-174, with *bla*CTX-M-15 as the major ESBL genotype. **Conclusions:** sUTIs in Oman show a high burden of ESBL-producing *E. coli*. Nitrofurantoin, mecillinam, fosfomycin, temocillin, and nitroxoline would be effective carbapenem-sparing oral options. Continuous phenotypic and genomic surveillance are crucial to guide antimicrobial therapy and stewardship.

## 1. Introduction

Clinically, UTIs range from uncomplicated cystitis (simple urinary tract infections (sUTIs)), typically presenting with dysuria, urinary frequency, urgency, and suprapubic discomfort, to acute pyelonephritis, which may manifest with fever, flank pain, and systemic features. Diagnosis relies on clinical presentation supported by urinalysis and is confirmed by urine culture with antimicrobial susceptibility testing.

sUTIs are among the leading causes of bacterial infections [1]. They represent a significant global health concern, particularly among women. In fact, approximately 50% to 60% of women will experience at least one UTI in their lifetime, with nearly 10% reporting an infection annually [2]. Moreover, these infections affect about 8% of pregnancies, posing serious risks such as pyelonephritis, preterm birth, and low birth weight if left untreated [3]. Among sexually active women aged 15 to 50 years, the incidence rate of uncomplicated cystitis is estimated at approximately 2.91 per 100 person-years [4]. Additionally, the prevalence of UTIs increases with age, reaching about 20% in women aged 65 years and older [5].

Although oral agents remain effective for many sUTIs, the increasing prevalence of multidrug-resistant (MDR) uropathogens, particularly extended-spectrum β-lactamase (ESBL)-producing *Escherichia coli*, compromises empiric therapy and drives the use of broader-spectrum antibiotics. This challenge is especially important in pregnancy, where asymptomatic bacteriuria and symptomatic infection are associated with higher risks of progression to pyelonephritis and adverse maternal–fetal outcomes if not detected and treated promptly.

Most of these sUTIs are primarily managed in outpatient settings, where a large volume of antibiotics is dispensed, often in a largely unregulated manner. This is partly because antimicrobial stewardship efforts have traditionally focused on inpatient care [6].

Epidemiologically, *E. coli* remains the predominant uropathogen in Oman and globally, followed by organisms such as *Klebsiella pneumoniae*, *Proteus mirabilis*, and *Enterococcus* spp., with regional variation in resistance patterns. Across the Middle East and the Gulf region, *E. coli* remains the most common causative agent of both community- and hospital-acquired UTIs. *There is growing global concern over the spread of multidrug-resistant (MDR) clones* [7], *with extended-spectrum β-lactamase (ESBL)–producing E. coli dominating clinical settings* [8]. Of particular concern, the ESBL determinant blaCTX-M-15 is widely disseminated, often via transferable plasmids, and is frequently linked to successful extraintestinal pathogenic *E. coli* lineages, including ST-38, ST-405, and ST-69, which have been reported across diverse geographic settings. High-risk clones such as ST-131 have been reported across several countries in the region, including Oman, Kuwait, Qatar, and Saudi Arabia, highlighting their widespread dissemination and association with resistant infections [9,10]. In Saudi Arabia, this clone is widespread and often carries the *bla*CTX-M-15 genotype [9]. Other frequently reported clones are ST-38, ST-405, and ST-69, which are linked to fluoroquinolone and β-lactam resistance. Recently, the United Arab Emirates (UAE) has reported the emergence of ST-1193, a highly resistant clone in younger women [11]. The convergence of virulence and resistance in such lineages increases the risk of treatment failure, limits oral options, and amplifies the public-health burden, highlighting the need for improved diagnostic and antimicrobial stewardship strategies.

The increasing prevalence of these MDR clones presents serious therapeutic challenges, which merit the introduction of newer antimicrobials. With the paucity of newer antimicrobials, nitroxoline (NI), mecillinam (MEC), temocillin (TMO), and fosfomycin (FOS) are gaining increasing traction due to their efficacy against MDR uropathogens in UTI [12].

Nitroxoline, an oral 8-hydroxyquinoline derivative, is currently prescribed in Germany, Poland, and Croatia, while mecillinam is commonly prescribed in Scandinavia and has recently been approved in the U.S. [13]. Temocillin is widely prescribed in the UK, Belgium, and France, targets MDR Gram-negative bacteria and is stable against ESBLs and AmpC enzymes, making it a practical carbapenem-sparing option [14]. Fosfomycin with single-dose oral therapy is suitable for uncomplicated UTIs against MDR pathogens [13].

This study explored the etiology, antimicrobial susceptibility profile, and prevalence of extended-spectrum β-lactamases (ESBL), AmpC β-lactamases (AmpC), and carbapenemases in women of reproductive age with UTI. Phenotypic detection of ESBLs and AmpCs was performed to assess the feasibility of conducting these tests in low-resource centers. Carbapenem resistance was assessed using complementary phenotypic and genomic approaches, rather than implying standalone phenotypic carbapenemase testing for all isolates. Finally, representative ESBL *E. coli* isolates were tested against NI, FOS, MEC, and TMO and subjected to whole genomic sequencing (WGS) to assess the circulating clones, resistance, and virulence gene profiles among the circulating uropathogenic strains.

## 2. Results

Most patients included in the study were from Sultan Qaboos University Hospital (SQUH), comprising 309 cases (41%). This was followed by healthcare centers affiliated with Al-Masarah Hospital with 230 patients (30%), Al-Nahda Hospital with 135 patients (18%), and Khoula Hospital with 88 patients (12%).

The distribution of uropathogens mirrored the patient’s distribution across these centers. SQUH yielded the highest number of isolates (342, 43%), followed by Al-Masarah (230, 29%), Al-Nahda Hospital (135, 17%), and Khoula Hospital (88, 11%). The slightly higher number of isolates from SQUH reflects multiple bacterial isolates recovered from some patients’ midstream urine (MSU) samples.

In total, 795 uropathogens were isolated from MSU cultures of sUTI patients across the four centers. Among the 14 identified species, *Escherichia coli* (n = 489, 62%) and *Klebsiella pneumoniae* (*K. pneumoniae*) (n = 140, 18%) were the predominant species. *E. coli* was the leading uropathogen in all centers, ranging from 8% in Khoula Hospital to 25% in SQUH, while *K. pneumoniae* exhibited variable prevalence, reaching its highest proportion (6.8%) at SQUH.

### 2.1. Microbial Etiology and Antimicrobial Susceptibility Profiles

Across all four centers, as seen in Table 1, *E. coli* isolates demonstrated a higher susceptibility rate to nitrofurantoin, averaging at 97.6%, in contrast to *K. pneumoniae* isolates, which exhibited a lower susceptibility rate of 82.3%. When considering both *E. coli* and *K. pneumoniae*, their susceptibility rates to cotrimoxazole were 66.4% and 82.1%, respectively. Similarly, their susceptibility to ciprofloxacin was 49.6% for *E. coli* and 59.3% for *K. pneumoniae*.

Comparatively, the susceptibility to cephalosporins was somewhat lower among *E. coli* isolates (cefazolin at 45.8%, cefuroxime at 53.5%, ceftriaxone at 61.24%, ceftazidime at 66.4%, and cefepime at 41.7%) when compared to *K. pneumoniae* isolates (cefazolin at 47.9%, cefuroxime at 73.1%, ceftriaxone at 82.3%, ceftazidime at 85.3%, and cefepime at 59.7%). Additionally, the susceptibility rate to amoxicillin-clavulanate in *E. coli* was 85.9%, while in *K. pneumoniae*, it was 66.2%.

*E. coli* isolates displayed higher susceptibility rates to piperacillin-tazobactam at 98.9% when compared to *K. pneumoniae* isolates at 85.6%. Furthermore, *E. coli* isolates exhibited high susceptibility rates to carbapenems (ertapenem, imipenem, and meropenem, all at 100%), aminoglycosides (amikacin at 99.2% and gentamicin at 89.8%), and ceftazidime-avibactam (99.3%). In contrast, *K. pneumoniae*’s susceptibility to carbapenems (ertapenem at 94.3%, imipenem at 99%, and meropenem at 98.5%), aminoglycosides (amikacin at 96.5% and gentamicin at 94.8%), and ceftazidime-avibactam (97.3%) was slightly lower than that of *E. coli*.

The antimicrobial susceptibility profiles of *E. coli* and *K. pneumoniae* across the four participating centers are summarized in Table 2. It’s important to note that all these percentages represent average susceptibilities from all four centers. Overall, carbapenems exhibited excellent activity, with both meropenem and imipenem demonstrating 100% susceptibility for *E. coli* in all centers and nearly complete activity against *K. pneumoniae* (95.9–100%). Amikacin also maintained high efficacy, with susceptibility ranging from 98.8% to 100% across sites. Piperacillin–tazobactam showed similarly strong performance (97.5–100%), while nitrofurantoin remained highly effective against *E. coli* (96.5–98.5%) but demonstrated lower activity against *K. pneumoniae* (73–78%).

Moderate susceptibility was observed for amoxicillin–clavulanate, particularly at Khoula Hospital (94.2%) and Al-Al-Nahdha Hospital (91.8%), with reduced rates at SQUH (79.3%) and Al-Masarrah Hospital (78.1%). Gentamicin displayed consistent activity (87.9–92.9%) across all centers. In contrast, the cephalosporins showed variable efficacy: ceftazidime ranged from 60.3% at Khoula Hospital to 77.6% at Al-Masarrah Hospital; ceftriaxone ranged from 59.6% to 61.8%; and cefazolin exhibited the lowest activity, especially at SQUH (15.6%), compared with 56–60% elsewhere. Cefepime susceptibility was moderate (30.4–63.6%), with the lowest value reported at Al-Masarrah Hospital.

Lower susceptibility rates were recorded for ampicillin (30.2–37.9%), cotrimoxazole (63.8–71.0%), and ciprofloxacin (58.6–64.2%) across all sites. Notably, ceftazidime–avibactam, tested exclusively at SQUH, displayed outstanding susceptibility (99.3%).

The prevalence of ESBL-producing *E. coli* varied among the four hospitals (Table 1). The highest prevalence was observed at Al-Masarrah Hospital, where 51.4% (73/142) of isolates were identified as ESBL producers. This was followed by SQUH, where 39.5% (79/200) of isolates exhibited the ESBL phenotype, and Al-Al-Nahdha Hospital, which showed a comparable rate of 39% (34/87). Khoula Hospital reported the lowest ESBL prevalence, with 38.3% (23/60) of isolates identified as ESBL producers.

Furthermore, AmpC β-lactamase-producing isolates were not detected in SQUH, Khoula, or Al-Masarrah Hospitals; however, four isolates (4/87; 4.6%) were identified at Al-Al-Nahdha Hospital.

Differences in resistance trends were observed between ESBL-producing and non-ESBL-producing strains. ESBL producers showed slightly reduced susceptibility to nitrofurantoin (*p* = 0.167), cotrimoxazole (*p* = 0.187), cefoxitin (*p* = 0.128), ampicillin (*p* = 0.083), amoxicillin/clavulanate, gentamicin, ciprofloxacin, and piperacillin/tazobactam compared to their non-ESBL counterparts. However, due to low number of isolates, the significance of this trends cannot be concluded.

### 2.2. Assessment of Various β-Lactam/β-Lactamase Inhibitors for ESBL Detection via the Double-Disk Synergy Test (DDST)

**Figure 1:**
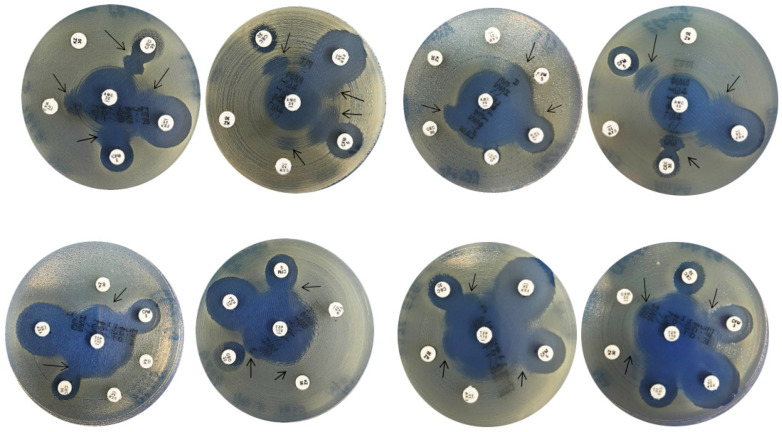
Double Disc Synergy Test (DDST). Detection of ESBL production in *E. coli* using several cephalosporins: cefepime (FEP), Cefpodoxime (PX), Ceftriaxone (CRO), Cefixime (CFM), and Cefaclor (CEC) against two different β-lactam/β-lactamase inhibitors: Amoxicillin-clavulanate (AMC) and Piperacillin/tazobactam (TZP). Black arrows indicate some synergistic pattern in the DDST.

ESBL production was phenotypically evaluated using the double-disk synergy test (DDST), a well-established confirmatory method for detecting clavulanate- or tazobactam-inhibitable β-lactamases. In this assay, disks containing selected extended-spectrum cephalosporins were placed at a standardized distance from disks containing β-lactam/β-lactamase inhibitor combinations (amoxicillin–clavulanate or piperacillin–tazobactam) on Mueller–Hinton agar. Enhancement or distortion of the inhibition zone of the cephalosporin disk toward the inhibitor disk was interpreted as positive synergy, indicating ESBL activity (Figure 1).

A total of 30 phenotypically confirmed ESBL-producing *E. coli* isolates were evaluated. The frequency of observable synergy varied according to the cephalosporin tested. Cefepime showed the highest rate of synergy (83%), followed by cefixime (79%) and cefaclor (75%), suggesting that these substrates are particularly sensitive for detecting inhibitor-reversible hydrolysis by ESBL enzymes in this isolate set. In contrast, ceftriaxone demonstrated moderate sensitivity (50%), whereas synergy was less frequently observed with cefuroxime (63%) and cefazolin (88%).

### 2.3. Antimicrobial Susceptibility to Nitroxoline, Fosfomycin, Mecillinam and Temocillin in ESBL E. coli

**Figure 2:**
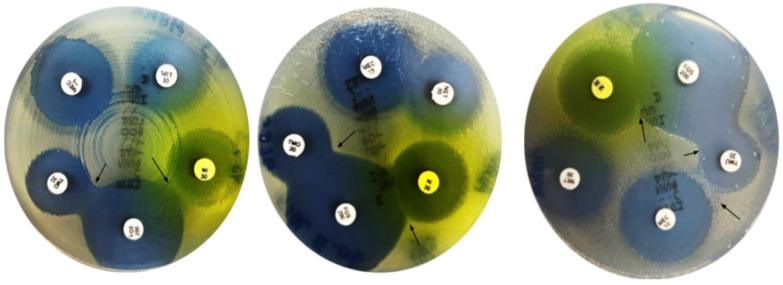
Susceptibility of *E. coli* to nitroxoline, fosfomycin, temocillin, mecillinam and netimicin. Note: Arrows indicate potentiation between fosfomycin and temocillin and antagonism (Flattening) between fosfomycin and nitroxoline.

Table 3 and Figure 2 summarize the antimicrobial susceptibility profiles of ESBL-producing and non-ESBL-producing *E. coli* isolates against mecillinam, temocillin, nitroxoline, and fosfomycin. Overall, all four agents demonstrated high in vitro activity against both isolate groups, with minimal differences observed between ESBL and non-ESBL phenotypes.

Mecillinam, nitroxoline, and fosfomycin showed complete activity, with 100% susceptibility among both ESBL-producing and non-ESBL-producing isolates. This uniform susceptibility across resistance profiles indicates that the activity of these agents remains largely unaffected by ESBL production, supporting their robustness against common β-lactamase-mediated resistance mechanisms in *E. coli*. The absence of resistant or intermediate isolates further highlights their potential reliability for empirical treatment of sUTIs.

In contrast, temocillin exhibited a modest reduction in activity among ESBL-producing isolates. While all non-ESBL isolates were fully susceptible (100%), susceptibility among ESBL producers was 89.3% (27/30), with the remaining 10.7% (3/30) categorized as intermediate. Notably, no temocillin resistance was observed in either group. This pattern suggests that, although temocillin retains substantial activity against ESBL-producing *E. coli*, its efficacy may be partially influenced by specific resistance mechanisms or strain-related factors present within the ESBL population.

### 2.4. Genotypic Characterization of ESBL Producing E. coli

The genetic diversity of ESBL-producing *E. coli* isolates was investigated using multi-locus sequence typing (MLST), a standardized genotyping method that classifies bacterial isolates into sequence types (STs) based on the allelic profiles of seven conserved housekeeping genes. Isolates sharing the same ST are considered closely related genetically and may represent successful lineages that circulate within communities or healthcare settings.

Among the 26 ESBL-producing *E. coli* isolates analyzed, 15 distinct sequence types were identified (Table 4), indicating a genetically diverse population rather than dominance by a single clone. Despite this diversity, several STs were repeatedly detected, suggesting the presence of locally prevalent or internationally disseminated lineages.

The most frequently identified sequence types were ST-131, ST-174, ST-1193, and ST-73, each represented by three isolates (11.5% per ST). Collectively, these four STs accounted for nearly half of all ESBL-producing isolates, highlighting their epidemiological relevance. Notably, ST-131 is a globally recognized high-risk clone commonly associated with ESBL production and multidrug resistance, while ST-1193 and ST-73 have increasingly been reported in sUTIs and are known for their pathogenic potential. The detection of ST-174, a less frequently reported lineage, suggests possible regional circulation within the study setting.

Several additional STs were identified at lower frequencies. ST-38, ST-141, and ST-127 were each detected in two isolates, whereas eight STs (ST-69, ST-7401, ST-12, ST-410, ST-998, ST-10, ST-44, and ST-69) were each represented by a single isolate, further emphasizing the heterogeneity of ESBL-producing *E. coli* in this cohort. The presence of both globally recognized and less common STs suggests multiple independent acquisition events rather than expansion of a single dominant clone. Importantly, a novel sequence type was identified in isolate EC 1286, indicating the emergence of a previously unreported genetic lineage.

**Figure 3:**
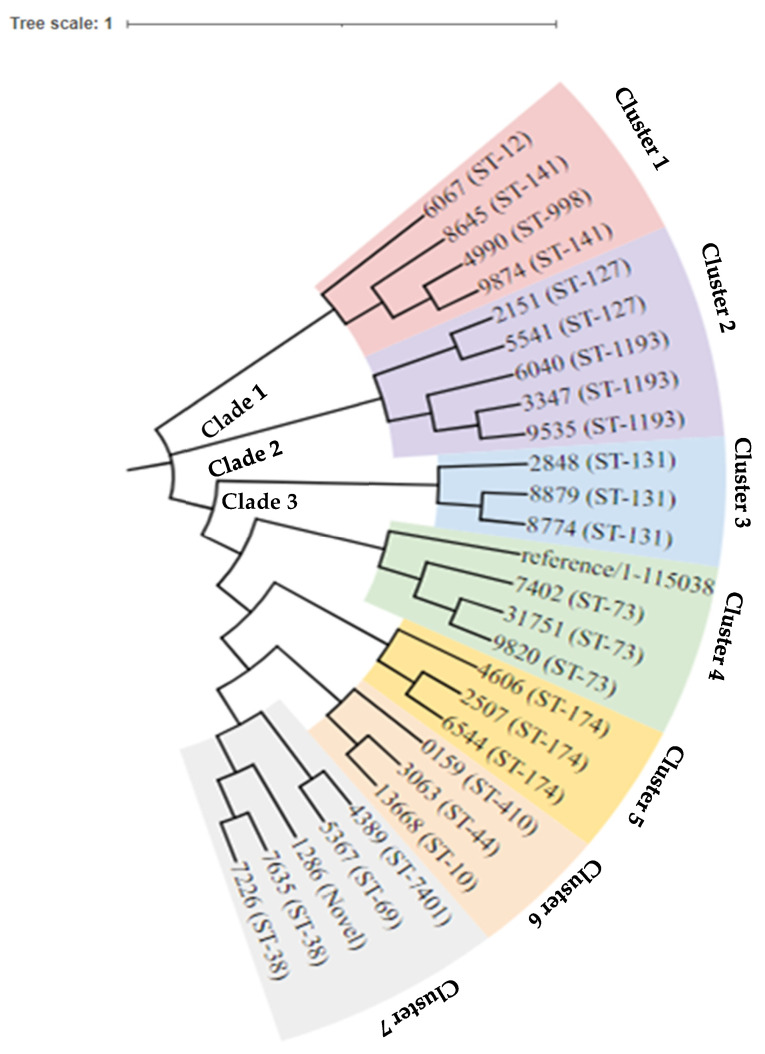
Phylogeny tree of the 26 strains of *E. coli* with three main clades.

The phylogenetic analysis revealed three distinct primary clades (Figure 3). Clade one comprised clusters featuring sequence types ST-12, ST-141, and ST-998. Clade 2 encompassed a solitary cluster with sequence types ST-127 and ST-1193. Clade 3 exhibited five principal clusters, including sequence types ST-410, ST-44, ST-10, ST-7401, ST-69, Novel-ST, and ST-38. Notably, isolates with ST-998, ST-12, and ST-141 demonstrated close relatedness, as did ST-69 and ST-7401. Lastly, isolates with ST-410, ST-44, and ST-10 also showed a discernible level of relatedness within the phylogenetic structure.

The distribution of key antimicrobial resistance genes across different *E. coli* sequence types varied. Amongst the ESBLs, *bla*CTX-M-15 predominated detected in 17 isolates, followed by *bla*CTX-M-14 and *bla*CTX-M-27 (4 and 3 isolates, respectively). *bla*TEM-1, was detected in four isolates as was blaOXA-1, was present in four isolates. The AmpC gene (DHA-1) was identified in two strains.

*bla*CTX-M-15, frequently co-occurred with *cpxA*. Many ESBL-producing strains also carried efflux pump regulators such as *emrR*, *emrB*, and *mdtH*, along with global regulators like *marA* which was present in 11 isolates. In addition, resistance determinants like *aadA5* (aminoglycoside resistance) and QnrS1 (fluoroquinolone resistance) were frequently present in ESBL strains. Genes such as *evgA* and H-NS were found universally across all isolates while *msbA* and *mdtN* had a more limited distribution.

Table 5 summarizes the distribution of efflux resistance genes identified among 26 *E. coli* isolates. A total of 13 different efflux-associated genes were detected, belonging to various efflux pump families, including RND (Resistance-Nodulation-Division), ABC (ATP-Binding Cassette), MFS (Major Facilitator Superfamily), and regulatory systems.

Among these, the *evgA* and H-NS genes were universally present in all isolates, indicating their conserved role in global regulation and stress response. The *marA* regulator, another well-known global transcriptional activator associated with multiple antibiotic resistance, was detected in 11 isolates (42.3%), making it the most frequent regulator after *evgA* and H-NS.

Other efflux-related genes were detected at lower frequencies. The *msbA* and *mdtN* genes (ABC and RND families, respectively) were each identified in three isolates (11.5%), while the *acrB–AcrE–mdtP* cluster was found together in two isolates (7.7%), suggesting coordinated expression of multidrug efflux systems within these strains.

Several genes were detected only once, including *yojI* (ABC family), PmrF (LPS modification system), *evgS* (two-component system), *acrD*, AcrS (both RND family members), and *emrA* (MFS family). The presence of these unique genes highlights the genetic diversity and heterogeneity of efflux-mediated resistance mechanisms among the isolates studied.

Table 6 summarizes the distribution of antimicrobial resistance genes detected in 26 *E. coli* isolates, categorized according to their respective multi-locus sequence types (MLSTs). The results reveal a wide range of resistance determinants spanning multiple antibiotic classes, with notable variability between sequence types.

Resistance genes associated with aminoglycosides were among the most frequently observed, including *aph(6)-Id*, *aph(3″)-Ib*, *aac(6′)-Ib-cr*, *aac(3)-IIa*, and *aadA* variants (*aadA1*, *aadA5*), commonly present in high-risk lineages such as ST-131, ST-174, and ST-69. The coexistence of multiple aminoglycoside-modifying enzymes in several isolates suggests strong selective pressure from aminoglycoside exposure.

For quinolone resistance, mutations in *gyrA* and the plasmid-mediated gene *qnrS1* were detected in a subset of isolates, particularly within ST-1193 and ST-131, two globally disseminated fluoroquinolone-resistant clones. These findings align with the phenotypic resistance typically associated with these sequence types.

Resistance to folate pathway antagonists was mediated by *sul1*, *sul2*, and *dfrA* (*dfrA1*, *dfrA14*, *dfrA17*) genes, with these determinants frequently co-occurring in isolates that also harbored aminoglycoside and β-lactamase genes, indicating the presence of multi-resistance plasmids.

Tetracycline resistance was conferred by *tet(A)* and *tet(B)*, detected in a limited number of isolates, while macrolide resistance was attributed to *mrx* and *mph(A)*, each identified sporadically.

Among β-lactamase genes, multiple classes were detected. The most prevalent ESBLs included *bla*CTX-M variants *(bla*CTX-M-14b, *bla*CTX-M-15, *bla*CTX-M-27), particularly within ST-131 and ST-1193. *bla*TEM-1B and *bla*SHV-12 were also detected, often co-carried with *bla*OXA-1, highlighting the multidrug-resistant potential of these clones. A single isolate harbored an AmpC (*bla*DHA-k) gene, whereas OXA-type β-lactamases were detected in a few isolates belonging to high-risk lineages.

Table 7 presents the distribution of virulence genes detected among 26 *E. coli* isolates representing 15 distinct STs. A wide range of virulence-associated factors were identified, encompassing genes involved in adhesion, iron acquisition, toxin production, capsule formation, and immune evasion, reflecting the genetic and functional diversity of the isolates.

Among adhesion-related genes, *fimH*, encoding type 1 fimbriae, was the most prevalent, detected in 13 isolates (50%), followed by *focG*, *papC*, and members of the *sfa* and *yeh* fimbrial clusters, each detected in fewer isolates. Genes linked to iron acquisition such as *fyuA*, *irp2*, *sitA*, and *iss* were widely distributed, each detected in up to 11 isolates, underscoring the importance of siderophore-mediated iron uptake in *E. coli* virulence.

Capsule-associated genes, including *kpsE*, *kpsMII*, and *neuC*, were also found in several isolates, suggesting that polysialic acid capsule synthesis may contribute to serum resistance and persistence in the urinary tract. Toxin-related genes, such as *hlyA*, *cnf1*, *usp*, *vat*, and *sat*, were present in a smaller subset (3–4 isolates), typically associated with ExPEC pathotypes.

Notably, high-risk global lineages such as ST-131, ST-1193, and ST-73 carried multiple virulence factors, particularly those involved in adhesion and iron acquisition, highlighting their pathogenic potential. Less common sequence types (e.g., ST-998, ST-410, ST-44) carried fewer virulence genes, indicating variability in virulence gene content across clonal lineages.

## 3. Discussion

Epidemiological studies estimate that approximately 50–60% of women in the reproductive age group (15–49 years) experience at least one UTI during their lifetime, and a significant proportion will have recurrent infections, due to a combination of anatomical, hormonal, behavioral, and microbiological factors. Anatomically, the shorter female urethra and its proximity to the anus facilitate ascending bacterial entry, particularly by uropathogenic *E. coli*. Hormonal fluctuations during the reproductive years, including menstrual cycling and pregnancy, influence the vaginal microbiota and may reduce protective *Lactobacillus* species, thereby increasing susceptibility to colonization. Sexual activity, contraceptive practices, hydration habits, and delayed voiding further contribute to UTI risk in this age group. Recurrent infections are additionally driven by the ability of uropathogens to adhere to uroepithelial cells, persist as intracellular bacterial communities, and re-emerge after treatment, as well as by host susceptibility and repeated antimicrobial exposure [15,16]. Among the reproductive group, pregnant women are particularly vulnerable due to physiological changes in the urinary tract, with UTIs occurring in 2–10% of all pregnancies [3]. In contrast, non-pregnant women in the same age range have a UTI prevalence ranging from 20–30%, depending on factors such as sexual activity, contraception use, and personal hygiene practices [17].

We evaluated the susceptibility profiles of *E. coli* and *K. pneumoniae* in pregnant and non-pregnant women to promote antimicrobial stewardship. In addition, we explored the interplay between AMR and virulence genes in whole-genome sequenced representative *E. coli* isolates. A study analyzed 1798 urine cultures and found that *E. coli* and *K. pneumoniae* were the predominant uropathogens, accounting for 60% and 33.2% of isolates, respectively. Notably, *E. coli* isolates exhibited high susceptibility to fosfomycin (98.6%) and nitrofurantoin (80%), whereas *K. pneumoniae* showed lower susceptibility to nitrofurantoin (17.2%) but remained relatively susceptible to fosfomycin (73.9%) and amikacin (50.2%) [18].

Although overall antimicrobial susceptibility did not differ significantly according to pregnancy status, wild-type *E. coli* isolates—defined as strains lacking acquired resistance mechanisms and exhibiting standard antibiotic susceptibility—were more frequently identified among pregnant women (31%) than non-pregnant women (24%) [18]. Similar patterns have been reported in previous studies, where lower rates of antimicrobial resistance among uropathogens isolated from pregnant women were observed compared with non-pregnant populations [19,20,21]. These authors attributed this trend to differences in healthcare exposure and antibiotic selection pressure. Pregnant women are more likely to undergo routine antenatal screening, enabling early detection and treatment of bacteriuria, often before repeated antibiotic exposure occurs [21]. In contrast, non-pregnant women may experience recurrent or self-managed UTIs, which are associated with higher cumulative antibiotic use and increased selection of resistant strains [19]. Moreover, antibiotic prescribing during pregnancy is typically more restricted and guideline-driven, which may further limit resistance development [20]. Similar observations have also been reported in studies from Europe and Asia, where ESBL-producing *E. coli* were less prevalent among pregnant women than among non-pregnant women [22,23], supporting the interpretation that pregnancy-related clinical practices may favor the persistence of wild-type strains.

Our study confirms that nitrofurantoin remains a reliable empirical option for the treatment of uncomplicated urinary tract infections (sUTIs), demonstrating high susceptibility rates in both pregnant (93.5%) and non-pregnant women (97.6%), along with favorable pharmacokinetic properties, including oral availability, urinary tract specificity, and minimal impact on the gut microbiota. Nitrofurantoin is active against a broad range of Gram-negative and Gram-positive uropathogens, including *E. coli*, supporting its role as a cephalosporin- and fluoroquinolone-sparing agent in sUTI management [24]. In non-pregnant women, nitrofurantoin is generally well tolerated, with the most commonly reported adverse effects being mild gastrointestinal symptoms such as nausea and abdominal discomfort; rare but recognized complications include pulmonary hypersensitivity reactions, hepatotoxicity, and peripheral neuropathy, particularly with prolonged or repeated use. In pregnancy, nitrofurantoin is considered safe during the first and second trimesters and is widely recommended in clinical guidelines; however, its use is generally avoided near term (after 38 weeks’ gestation) and during labor due to the risk of neonatal hemolytic anemia, especially in infants with glucose-6-phosphate dehydrogenase (G6PD) deficiency. Despite these considerations, when appropriately prescribed, nitrofurantoin offers a favorable balance between efficacy and safety, reinforcing its value as a first-line therapy for sUTI while reducing reliance on broader-spectrum antibiotics [25,26,27].

Moderate susceptibility rates (50–80%) observed for fluoroquinolones, cephalosporins, and cotrimoxazole reflect the increasing impact of well-characterized genetic resistance mechanisms identified through WGS. Reduced susceptibility to fluoroquinolones is commonly associated with chromosomal point mutations in the quinolone resistance-determining regions (QRDRs) of the *gyrA* and *parC* genes, which encode DNA gyrase and topoisomerase IV, respectively. In addition, WGS frequently detects plasmid-mediated quinolone resistance (PMQR) genes, such as *qnr* variants and *aac(6′)-Ib-cr*, which confer low-level resistance but facilitate the selection of higher-level resistance when combined with chromosomal mutations. The presence of these determinants explains the intermediate susceptibility patterns observed in this study and is consistent with global reports linking fluoroquinolone resistance to prior antibiotic exposure and horizontal gene transfer [28,29].

For cephalosporins, reduced susceptibility is primarily driven by the presence of ESBL genes, particularly *blaCTX-M* variants (notably *blaCTX-M-15*), as well as *blaTEM* and *blaSHV*. These enzymes hydrolyze third- and fourth-generation cephalosporins, leading to diminished activity despite in vitro susceptibility in some isolates. Additional mechanisms, including porin loss or alteration and increased expression of efflux pumps, can further reduce cephalosporin permeability and enhance resistance, particularly in ESBL-producing strains. Similar genetic profiles have been widely reported in uropathogenic *E. coli* from both community and hospital settings.

Reduced susceptibility to cotrimoxazole (trimethoprim–sulfamethoxazole) is typically associated with the acquisition of dihydrofolate reductase (*dfr*) genes conferring trimethoprim resistance and sulfonamide resistance genes (*sul1*, *sul2*), which encode altered dihydropteroate synthase. These genes are often carried on integrons and plasmids, facilitating their spread among uropathogens and explaining the persistence of moderate susceptibility rates despite reduced clinical use in some regions.

When compared with other studies, our findings align with global trends showing declining susceptibility to these historically first-line agents. However, these findings contrast with previous studies that described slightly higher susceptibility rates to cephalosporins and fluoroquinolones [30,31,32]. Such differences likely reflect regional variation in antibiotic prescribing practices, local resistance gene pools, and clonal population structures, as revealed by WGS. Together, these results emphasize the value of integrating genomic data with phenotypic susceptibility testing to better understand resistance mechanisms and to inform context-specific empirical treatment guidelines.

Excellent activity was observed against aminoglycosides and piperacillin-tazobactam, making them effective carbapenem-sparing alternatives against ESBL-producing uropathogens.

ESBLs present a formidable challenge in healthcare settings, imparting resistance to multiple antibiotics and thereby limiting treatment options. The differences in susceptibility profiles between ESBL and non-ESBL producers underscore the importance of being aware of local ESBL rates when prescribing antibiotics.

Carbapenems, with 100% susceptibility, underscore their potency against extended-spectrum β-lactamase (ESBL) and AmpC-producing isolates. However, their use should be restricted to prevent the acceleration of carbapenem resistance [33]. It is essential to identify carbapenem-sparing options for managing ESBL-producing uropathogens. Notably, our study also demonstrated 100% susceptibility to nitroxoline among 30 ESBL-producing *E. coli* isolates, consistent with findings from a larger study of 394 multidrug-resistant *Enterobacterales* urinary isolates, where nitroxoline showed the highest activity: 99% susceptibility among ESBL producers, 98% among AmpC, and 100% among carbapenemase-producing and ESBL + AmpC isolates.

Fosfomycin is an excellent option for sUTIs as an empirical choice, as it has a broad spectrum of activity against both Gram-negative and some Gram-positive bacteria and retains efficacy in multidrug-resistant isolates. Importantly, in our study, *E. coli* isolates showed 100% susceptibility to fosfomycin. Its ability to achieve high concentrations in the urinary tract enhances its effectiveness in treating UTIs. The single-dose oral formulation improves patient compliance and is a safe choice during pregnancy. Fosfomycin’s unique mechanism of action reduces the likelihood of cross-resistance with other commonly used antibiotics [31]. Prior studies have demonstrated that fosfomycin and nitrofurantoin remain appropriate agents for the empirical management of sUTI [32,33,34,35].

Mecillinam represents a valuable empiric option for sUTIs due to its high and selective activity against *E. coli*, including many ESBL-producing strains, and its minimal impact on the intestinal microbiota [12,13]. This limited microbiota disruption is primarily explained by the drug’s pharmacokinetic properties: after oral administration as its prodrug pivmecillinam, the active compound is rapidly absorbed and preferentially excreted into the urine, resulting in low intestinal exposure and reduced selective pressure on commensal gut bacteria. In addition, mecillinam is a narrow-spectrum β-lactam with minimal activity against anaerobes and many non-target Gram-negative organisms that dominate the gut microbiota, further limiting ecological disturbance.

The strong activity of mecillinam against *E. coli* is attributed to its unique mechanism of action, as it selectively binds to penicillin-binding protein 2 (PBP2), which plays a critical role in maintaining the rod shape and cell wall integrity of *E. coli*. This target is distinct from those of many other β-lactams, and importantly, most ESBL enzymes do not efficiently hydrolyze mecillinam, allowing the drug to retain activity against ESBL-producing isolates. Furthermore, resistance to mecillinam typically requires specific chromosomal mutations affecting PBP2 or associated pathways, which occur relatively infrequently and often impose a fitness cost on the bacterium, limiting widespread dissemination.

The oral availability of pivmecillinam enhances its practicality for outpatient therapy and it is considered safe for use during pregnancy [36]. Consistently high susceptibility rates exceeding 95% across multiple European surveillance studies [37,38], along with guideline endorsement in several countries [39,40], support its role as a narrow-spectrum, carbapenem-sparing agent. In line with these reports, all *E. coli* isolates in the present study demonstrated 100% susceptibility to mecillinam, reinforcing its suitability for empirical treatment of sUTIs in settings facing increasing resistance to broader-spectrum antibiotics.

Temocillin is an effective oral agent for managing ESBL UTIs [39]. Its stability against ESBL and AmpC enzymes makes it a good carbapenem-sparing agent [40]. In our study, 89.3% of *E. coli* isolates were susceptible to temocillin, supporting its utility in treating ESBL-related UTIs. EUCAST breakpoints recommend high-exposure dosing (2 g q8h), which achieves urinary concentrations well above clinical MICs [39,40].

The MLST analysis revealed substantial genetic diversity among *E. coli* isolates, with 15 distinct sequence types (STs) identified, indicating that UTIs in this population are caused by multiple unrelated lineages rather than a single dominant clone. Such diversity is commonly reported in sUTIs and reflects the broad ecological reservoir of *E. coli*, ongoing horizontal gene transfer, and repeated introduction of strains from different sources. Despite this heterogeneity, the predominance of ST-131, ST-1193, ST-73, and ST-174 suggests selective expansion of well-adapted lineages with enhanced capacity for colonization, persistence, and, in some cases, antimicrobial resistance. ST-131 and ST-1193 are globally disseminated high-risk clones frequently associated with ESBL production and fluoroquinolone resistance, and their dominance has been widely reported in studies from Europe, Asia, and the Middle East, supporting the global success of these lineages. ST-73, in contrast, is typically linked to extraintestinal pathogenicity rather than extensive resistance, indicating that virulence, in addition to resistance, contributes to clonal success. The presence of ST-174, which has been less frequently reported in international studies, may reflect regional circulation or local selective pressures, such as antibiotic prescribing patterns. Compared with studies reporting clonal dominance by one or two STs, the broader ST distribution observed here suggests a dynamic population structure, emphasizing the importance of local genomic surveillance to capture regional differences in circulating uropathogenic *E. coli* and to inform empirical treatment and public health strategies [41].

ST-131, the leading sequence type is widely recognized for its pandemic multidrug-resistant lineage, often associated with the extended-spectrum β-lactamase gene *bla*CTX-M-15. It is frequently isolated from urinary tract and bloodstream infections in both community and hospital settings and combines both antimicrobial resistance and virulence traits [41].

The prevalence of ST-1193, which clusters with ST-127, is increasing globally, and its presence in our study suggests it may be gaining a foothold in local populations. It is primarily associated with fluoroquinolone resistance and has increasingly been detected in clinical isolates worldwide. Emerging evidence indicates that ST-1193 is increasingly supplanting ST-131 in certain regions, with respect to both prevalence and antimicrobial resistance, particularly among uropathogenic *Escherichia coli* strains [42]. 

The identification of ST-73 in this study reflects the continued circulation of a *E. coli* lineage characterized by high virulence but relatively low antimicrobial resistance, which is typical of community-acquired extraintestinal pathogenic *E. coli* (ExPEC). The pathogenic potential of ST-73 is largely driven by the presence of key virulence genes, particularly *pap*, *kpsMII*, and *fyuA*. The *pap* gene cluster encodes P fimbriae that mediate strong adhesion to uroepithelial cells, facilitating colonization of the urinary tract and ascent to the kidneys, and is therefore closely associated with symptomatic and severe UTIs. The *kpsMII* gene is involved in the synthesis of a group 2 polysaccharide capsule, which enhances resistance to host immune defenses such as complement-mediated killing and phagocytosis, promoting persistence and invasiveness. The *fyuA* gene encodes the receptor for the yersiniabactin siderophore system, enabling efficient iron acquisition in the iron-limited urinary environment and contributing to bacterial survival and competitiveness. Together, these virulence determinants act synergistically to enhance adhesion, immune evasion, and nutrient acquisition, explaining the high pathogenic potential of ST-73 despite its generally lower resistance profile [43].

While ST-174 is not as widely reported in the literature, its identification as a predominant sequence type in this study may indicate regional clonal expansion or a niche-specific adaptation. A study [44] recently reported the presence of ST-174 in neonatal sepsis cases in China, where it was associated with virulence traits including siderophore production and capsule expression. The emergence of ST-174 in this cohort suggests that previously underreported clones may be gaining clinical significance in specific geographical areas.

While mapping the epidemiology of UPEC isolates, circulating clades, and evolving resistance, efforts to assess treatment modalities go a long way in informing appropriate empirical management [45]. It was reported [46] that Fosfomycin resistant ESBL *E. coli* isolates belonged to the epidemic clone ST-131. Management of ST-1193 is more difficult. A study [47] reported that ST-69 (15%) and ST-131 (8%) predominated among UPEC isolates in Australia and were associated with 57% resistance to trimethoprim-sulfamethoxazole.

Beyond efflux-mediated resistance, antibiotic inactivation emerged as the second most prevalent resistance mechanism, driven largely by the acquisition of β-lactamase-encoding genes. A total of 15 distinct acquired genes were implicated in this mechanism amongst which five ESBL genes CTX-M-14, CTX-M-15, CTX-M-27, TEM-1 and SHV-12, predominated, with CTX-M-15 being the most common (65%), reaffirming its global role as a high-risk resistance determinant and SHV-12 the less prevalent (4%) [48]. The OXA-1 gene was found in five isolates. In addition, the co-production of both AmpC and ESBL was particularly detected in nine strains. Authors [48] reported that 31% of ESBL-producing *E. coli* strains carried at least one of the β-lactamase genes (*bla_CTX-M_*, *bla_TEM_*, *bla_SHV_*) in Iranian outpatients with UTI. A study from Nepal [46] utilized purified DNA from *E. coli* isolates as a template to detect ESBL genotypes, including *CTX-M*, *TEM*, and *SHV β*-lactamase genes. A study conducted in India investigated the coexistence of ESBL genes with carbapenemase, AmpC, and aminoglycoside resistance genes among uropathogens, highlighting *bla*_CTX-M-15_ as the most prevalent ESBL gene. Furthermore, it was found that ESBL genes co-existed with carbapenemase genes (*bla*_NDM-5_ and *bla*_OXA-48_), AmpC genes (*bla*_CIT_ and *bla*_DHA-1_), and aminoglycoside resistance genes (*rmtB*, *rmtA*, *rmtC and armA*) [49]. A recent study from Croatia reported that *i*n *E. coli* and *K. pneumoniae*, the dominant resistance mechanisms are ESBLs belonging to the CTX-M, TEM, and SHV families; p-AmpC; and carbapenemases belonging to classes A, B, and D [50]. Different research groups reported the detection of OXA-1 in *E. coli* isolates. The resistance genes blaCTX-M-15, blaNDM-5, blaOXA-48, blaCIT, blaDHA-1, OXA-1, rmtA, rmtB, rmtC, and armA do not directly encode classical virulence factors, but they have a major indirect impact on pathogenicity, clinical outcomes, and public health by severely limiting effective treatment options and promoting bacterial persistence and spread. blaCTX-M-15 confers resistance to third-generation cephalosporins and is widely associated with successful high-risk clones, leading to frequent empiric treatment failure and increased use of broader-spectrum antibiotics. blaNDM-5 and blaOXA-48 encode carbapenemases that compromise last-line β-lactams, markedly increasing morbidity, mortality, and outbreak potential in healthcare and community settings. Plasmid-mediated AmpC genes (blaCIT, blaDHA-1) further reduce cephalosporin efficacy and complicate laboratory detection, while OXA-1 can undermine β-lactam/β-lactamase inhibitor combinations, limiting carbapenem-sparing strategies. The 16S rRNA methyltransferase genes (rmtA, rmtB, rmtC, armA) confer high-level resistance to aminoglycosides, eliminating an important option for severe and combination therapy. Collectively, these genes increase the clinical impact of infections by delaying appropriate therapy, prolonging infection and colonization, facilitating horizontal gene transfer via mobile genetic elements, and driving the emergence and dissemination of multidrug- and carbapenem-resistant strains, representing a significant threat to both individual patient outcomes and public health [51,52,53].

The *marA* gene—known for enhancing efflux pump activity and reducing membrane permeability—was detected in 11 isolates, making it one of the most frequently encountered efflux-associated genes [54]. Overall, efflux mechanisms emerged as the dominant resistance strategy, with nearly 20 different genes contributing to this class. The universal presence of genes like *evgA* and H-NS suggests a conserved role in baseline efflux regulation.

Moreover, six efflux genes—YojI, PmrF, evgS, acrD, AcrS, and emrA—were identified as unique, each occurring in only one isolate, which may reflect isolate-specific adaptations or rare resistance elements.

Plasmid-mediated resistance is a significant concern, as drug resistance genes can spread rapidly and efficiently between bacterial species through horizontal gene transfer [55]. Among the seven plasmids detected Col (BS512), IncFII (pRSB107), IncFIA, IncFII, IncFIB (AP001918), IncFIB (H89-PhagePlasmid) and IncFIB (K), Col(BS512) (31%) and IncFII (pRSB107) (27%) predominated. A recent study in Oman reported the detection of IncFIA, IncFII, IncY, IncI1-I(Alpha), and IncX in *E. coli* [56]. IncF, IncFII plasmids are known carriers of broad spectrum of antibiotic resistance genes in *E. coli* [57]. Another study reported that most of the *E. coli* isolates carried IncFIA, IncFII, IncFIB, Col(BS512), IncL1, IncX3, and IncH plasmids [58].

Antibiotic efflux was the most common mechanism of resistance, with nearly 20 genes being implicated. In particular, the *marA* gene, responsible for antibiotic efflux and reduction of cellular permeability to antibiotics, was detected in 11 *E. coli* isolates. The efflux genes belonged to several efflux families, like resistance-nodulation-cell division (RND), ATP-binding cassette (ABC) antibiotic efflux pumps, and major facilitator superfamily (MFS) [59,60]. Antibiotic inactivation, coded by 15 acquired genes, was predominantly mediated by β-lactamase enzymes (EC-5, ampC beta-lactamase, TEM-1 and CTX-M genes) [61]. Antibiotic target replacement was mediated by *sul1*, *sul2*, *drfA14*, *drfA17*, *and PmrF.*

The identification of multiple efflux pump genes and plasmid replicons in *E. coli* isolates has important implications for human and public health, as these mechanisms contribute to multidrug resistance, reduced treatment efficacy, and the dissemination of resistance within communities and healthcare settings. Although several efflux genes (*yojI*, *pmrF*, *evgS*, *acrD*, *acrS*, and *emrA*) were detected only once, their presence suggests isolate-specific adaptive responses to antimicrobial pressure. More importantly, the detection of marA in multiple isolates is clinically significant, as this global regulator enhances antibiotic efflux while simultaneously decreasing outer membrane permeability, resulting in broad, low-level resistance across multiple drug classes and facilitating the selection of additional resistance mechanisms. The predominance of IncF-family plasmids (IncFIA, IncFII, IncFIB) and Col(BS512) further amplifies this risk, as these plasmids are well-established vehicles for horizontal gene transfer and are frequently associated with ESBL, AmpC, and other resistance determinants, a pattern consistent with reports from Oman and other regions. The co-occurrence of efflux-mediated resistance, plasmid-borne genes, and enzymatic mechanisms such as β-lactamase production and target replacement increase the likelihood of multidrug-resistant infections, delayed effective therapy, prolonged colonization, and onward transmission, highlighting the need for integrated genomic surveillance to inform antimicrobial stewardship and public-health interventions [62].

A total of 57 virulence factors (VFs) were identified in this study, with the highest prevalence observed in the ST-73 and ST-998 lineages. This observation aligns with findings from other studies that have consistently highlighted ST-73 as a high-virulence clone associated with extraintestinal pathogenic *E. coli* (ExPEC). In a large-scale study on community-acquired UTIs, ST-73 was reported as the predominant clone in male patients (50%) and the second most frequent in females (12%), exhibiting the highest virulence score among tested clones, with a median and mean score of 9 [63]. The virulence of ST-73 is attributed to the presence of multiple factors, including adhesins (*fimH*, *pap*), siderophores (*fyuA*, *irp2*), and protectins such as *iss*, which collectively enhance colonization and immune evasion in the urinary tract.

While ST-998 has been less frequently reported in the literature, ST-73 was similarly prevalent in this analysis suggests a noteworthy virulence profile. The presence of numerous virulence genes in ST-998 indicates its potential for causing extraintestinal infections, although its full pathogenic capacity requires further investigation. Recent molecular surveillance studies have begun to detect ST-998 in clinical settings, suggesting an emerging role in UTI epidemiology and warranting closer attention [42]. The detection of ST-998 with a high VF load in this study may represent a localized clonal expansion or an underrecognized lineage with evolving clinical significance.

The ability of *E. coli* to cause infections relies on its arsenal of VFs, particularly those that mediate attachment to host tissues. Among the most prevalent attachment genes identified in the isolates was *fimH*, which encodes the adhesin component of Type 1 fimbriae [63]. This gene was universally detected in all isolates, supporting its critical role in urinary tract colonization. According to a study [64], *fimH* is strongly associated with adhesion to uroepithelial cells and is a key determinant in cystitis and pyelonephritis. In *E. coli O159*, the entire YHD fimbriae cluster—comprising *yehA*, *yehC*, and *yehD*—was present. These genes encode an outer membrane lipoprotein, a chaperone, and a major pilin subunit, respectively, and are implicated in biofilm formation and epithelial attachment [64]. Additionally, *IpfA*, encoding long polar fimbriae, was also exclusive to *E. coli O159* and has been linked to intestinal colonization and interaction with Peyer’s patches [65]. Other notable fimbrial genes included *papA_F43* in *E. coli 31751*, *papA_F11* and *papA_fsiA_F16* in *E. coli 9535*—all of which code for P fimbriae pilin subunits. These fimbriae are critical for adhesion to kidney tissues and are frequently associated with upper urinary tract infections [66,67].

Another central virulence mechanism involves iron acquisition, a vital process given the iron-limited environment of the human host. Several siderophore-associated genes were found in subsets of isolates. *fyuA*, encoding the yersiniabactin receptor, facilitates iron uptake and is associated with enhanced virulence and stress response. Banerjee et al. (2023) showed that *fyuA* is commonly present in multidrug-resistant uropathogenic *E. coli* (UPEC) strains. *irp2*, also detected in this study, is essential for yersiniabactin biosynthesis and has been linked to bloodstream infections [68]. Furthermore, *chuA*, which encodes a hemin receptor, allows *E. coli* to scavenge iron from hemoproteins and has been correlated with survival in urine [69]. The *sitA* gene, encoding an iron/manganese transporter, enhances intracellular survival by aiding in oxidative stress resistance, as noted by Ahmad et al. [70]. Together, these genes illustrate the diversity of siderophore systems employed by *E. coli* to overcome host nutritional immunity.

In terms of resistance to serum bactericidal activity, *iss* (increased serum survival) was found in multiple isolates. This gene enhances bacterial resistance to the complement system, contributing to the development of bloodstream infections and sepsis. It has been reported [71] that high prevalence of *iss* in invasive *E. coli* infection, including strains responsible for neonatal meningitis. Additionally, *kpsMII_k23*, which was uniquely present in *E. coli 7402*, encodes a polysialic acid capsule transport protein. This component of the Group 2 capsule facilitates immune evasion by protecting against phagocytosis and complement-mediated lysis [72]. These genes contribute to systemic virulence, particularly in extraintestinal infections.

Although classical cytotoxins such as *hlyA* (hemolysin) or *cnf1* (cytotoxic necrotizing factor) were not detected, the isolates showed the presence of *mchC* and *mchF* in *E. coli 5367*, which are part of the microcin H47 operon. *mchC* is involved in the biosynthesis of the microcin peptide, while *mchF* encodes an ABC transporter that exports it. These microcins act as bacteriocins, inhibiting competing bacteria and enhancing niche colonization. Another study [73] highlighted the ecological advantage conferred by such systems during intestinal colonization, especially in mixed microbial environments.

The findings of the current study align with recently reported data on VFs in *E. coli* for UTIs. It was reported that several VFs such as P fimbriae (*pap*), type1 fimbriae, afimbrial adhesin I (*afaI*), hemolysin (*hly*), cytotoxic necrotizing factor 1 (*cnf 1*), aerobactin (*aer*), S fimbriae (*sfa*), adhesins, fimbriae, *kpsMT*, *ompT*, *usp*, *iroN*, *iha*, *set 1*, *astA*, group II capsule synthesis; *sfa/foc*, S and F1C fimbriae; *iutA*, *traT*, serum resistance; and *fimH* were identified in *E. coli* isolates causing UTIs [65,66]. In addition, a study [33] assessed the genetic relation and screening of VFs among carbapenemase- producing *E. coli* from UTI and found that the predominant virulence genes included *iutA* (97.66%), *fyuA* (85.33%), *inh* (83%), *traT* (82.33%), *papП* (96, 32%), *fimH* (93, 31%) and *csgA* (30.66%). Another study reported that the *papG* class II gene plays a critical role in the development of *E. coli* for UTIs, and *fimH* adhesion plays a role by acting synergistically with P *papG* class II adhesion [67].

## 4. Materials and Methods

### 4.1. Study Period

This multicentric study was conducted from September 2022 to August 2023 in the Department of Microbiology and Immunology, College of Medicine and Health Sciences, Sultan Qaboos University (SQU), Muscat, Oman, in collaboration with the Diagnostic Microbiology Laboratory at Sultan Qaboos University Hospital (SQUH). Clinical specimens were sourced from four centers—SQUH and the referring Khoula, Al Al-Nahdha, and Al-Masarrah Hospitals—with selected isolates processed and characterized at the SQUH Microbiology Diagnostic Laboratory for in-depth analysis. In total, 762 patients were included across these four centers. Ethical approval was granted by the Medical Research Ethics Committee (MREC) of the College of Medicine and Health Sciences, SQU (SQU-EC/377/2021).

The study assessed the demographic characteristics and antimicrobial susceptibility profiles of *E. coli* and *K. pneumoniae* isolated from adult females in the reproductive age group (15–50 years).

Eligible participants were women of reproductive age who met predefined clinical and microbiological criteria to ensure the study population represented sUTIs.

Inclusion criteria comprised two groups. First, non-pregnant women presenting with symptoms suggestive of acute uncomplicated UTI, including dysuria, urinary urgency, and increased frequency, and with no known structural, functional, or metabolic abnormalities of the urinary tract. Second, pregnant women, both symptomatic and asymptomatic, who were diagnosed with UTI during routine antenatal care, including cases of asymptomatic bacteriuria detected through screening, were included to capture the full spectrum of UTI presentations during pregnancy.

Exclusion criteria were applied to minimize confounding factors that could influence antimicrobial susceptibility and clinical outcomes. Women were excluded if they had complicated UTIs, defined as infections associated with anatomical abnormalities, urinary obstruction, indwelling catheters, renal disease, or immunocompromising conditions. Patients who had received systemic antibiotic therapy within the preceding four weeks were excluded to avoid transient suppression of bacterial growth or selection bias toward resistant strains. In addition, women with significant comorbidities (such as diabetes mellitus, chronic kidney disease, or immunosuppressive disorders) and post-menopausal women were excluded, as these conditions are associated with altered UTI pathophysiology and resistance patterns distinct from those seen in women of reproductive age.

### 4.2. Bacterial Identification and Susceptibility Testing

All bacterial samples included in this study were collected, transported and processed as per standard guidelines for diagnostic purposes. None of the samples included in this study was collected exclusively for the purpose of the study. Only samples meeting the inclusion criteria were recruited for the analysis. All samples were sub-cultured in CLED agar (Oxoid, Basingstoke Hampshire, UK) including the samples transported from other hospitals, prior to preservation in sterile CryoBeads (Mast Diagnostics, Derby, UK) at −80 °C for further analysis. Bacterial identification was conducted using the Phoenix™ automated system (BD Diagnostics, Sparks Glencoe, MD, USA) and MALDI-TOF MS (Bruker, Bremen, Germany) at SQUH, in accordance with standard guidelines [15]. Antimicrobial susceptibility testing was performed and interpreted according to CLSI M100: Performance Standards for Antimicrobial Susceptibility Testing, 32nd ed. (2022) [74]. The automated system identified ESBL producers, while the presence of AmpC was initially estimated from the susceptibility profiles.

### 4.3. Phenotypic Detection of ESBL and AmpC

Thirty representative *E. coli* isolates previously validated as producers of extended-spectrum beta-lactamase (ESBL) through the BD Phoenix automated systems, were subjected to phenotypic detection of ESBL and AmpC. From the total cohort of 489 *E. coli* isolates, subsets were selected using a purposeful stratified approach to ensure representativeness of the overall population. For expanded antimicrobial susceptibility testing, 30 ESBL-producing and 82 non-ESBL isolates were chosen to capture diversity in resistance phenotypes, clinical sources, and collection periods, while minimizing over-representation of clonally similar strains.

**ESBL Detection:** Double-disk synergy test was used for detection of ESBLs. Two beta-lactam/beta-lactamase inhibitors (BL/BLI), piperacillin/tazobactam (TZP,110 µg) and amoxicillin/clavulanic Acid (AMC, 30 µg) (Oxoid, Basingstoke, UK), were utilized to detect ESBLs. The BL/BLIs were placed at the center of the Mueller Hinton agar plates and cefepime (FEP, 30 µg) (Oxoid, Basingstoke, UK), cefixime (CFM, 5 µg) (Oxoid, Basingstoke, UK), cefuroxime (CXM, 30 µg) (Oxoid, Basingstoke, UK), cefazolin (KZ, 30 µg) (Oxoid, Basingstoke, UK), cefaclor (CEC, 30 µg) (Oxoid, Basingstoke, UK), and ceftriaxone (CRO, 30 µg) (Oxoid, Basingstoke, UK) were placed at a distance of 20 mm edge to edge from them. The plates were incubated at 35 °C ± 2 °C for 18–24 h and examined for the potentiation of zones of inhibition between the BL/BLIs and the cephalosporins. An isolate is classified as an ESBL producer when the inhibition zone around the combination disc exceeds that of the cephalosporin-only disc by ≥5 mm (Figure 1).

**AmpC Detection:** Disk approximation test was employed to detect AmpC beta-lactamases. Imipenem (IPM, 10 µg), cefoxitin (FOX, 30 µg), amoxicillin-clavulanate (AMC, 30 µg), and piperacillin-tazobactam (TZP, 110 µg) were used as inducers and ceftazidime (CAZ, 30 µg) as a substrate in this test. The center-to-center distance between antibiotic disks was kept at 25 mm. The plate was incubated at 35 °C ± 2 °C for 18–24 h. The flattening or blunting of the zone of inhibition between the inducers and ceftazidime was evaluated the next day. A flattened or indented zone of inhibition (a “D” shape) around the cephalosporin disk, specifically on the side facing the inducer disk is considered positive for AmpC. This indentation shows the inducer (Imipenem and Cefoxitin) triggers the production of AmpC, which then diffuses out and inactivates the nearby cephalosporin, reducing its inhibition zone (Figure 1).

### 4.4. Antimicrobial Susceptibility Testing of Fosfomycin, Nitroxoline, Mecillinam, Temocillin

Representative 30 isolates of ESBL-producing *E. coli* in addition to 82 non-ESBL producing *E. coli* were selected for further investigation. The analysis of 30 representative ESBL-producing *E. coli* isolates was considered sufficient to address the study objectives while balancing feasibility and resource-intensive downstream analyses, such as molecular characterization. Similar sample sizes have been widely reported in ESBL epidemiological and genomic studies. These selected isolates were tested against temocillin (TMO, 30 µg) (Liofilchem, Roseto degli Abruzzi, Italy), fosfomycin (FOS, 200 µg) (LIOFILCHEM, Italy), nitroxoline (NI, 30 µg) (LIOFILCHEM, Italy), and mecillinam (MEC, 10 µg) (LIOFILCHEM, Italy) by the Kirby-Bauer disk diffusion method as follows. Three to five bacterial colonies from overnight pure cultures from CLED agar were suspended in normal saline (Fisher Chemical, Cramlington, UK) and adjusted to a 0.5 McFarland standard (approximately 1–2 × 108 CFU/mL) using a CrystalSpec nephelometer (BD Diagnostics, Sparks Glencoe, MD, USA), according to the manufacturer’s recommendations. After 15 min, the suspension was spread onto a Mueller–Hinton agar (MHA) surface (Oxoid, Hampshire, UK) and left for 1–2 min at room temperature to be absorbed. After 15 min, the selected antibiotic disks were placed on the inoculated MHA plates using sterile forceps. The plates were incubated at 37 °C for 18–24 h. Interpretation was based on the guidelines of the CLSI (2022) and the European Committee on Antimicrobial Susceptibility Testing for temocillin (Figure 1).

### 4.5. Genomic DNA Extraction and Purification and Whole Genome Sequencing (WGS)

Genomic DNA was extracted from 26 *E. coli* isolates (twenty-three ESBL producers and three wild-type strains). For whole-genome sequencing, 26 isolates (23 ESBL-producing and 3 non-ESBL) were selected based on phenotypic heterogeneity, resistance profiles, and temporal distribution to reflect the genetic diversity of ESBL-producing within the cohort. Isolates were drawn from multiple settings, reducing sampling bias and supporting generalizability of the genomic findings.

The DNA was extracted using Qiagen kit (QIAamp^®^ genomic DNA kit, Hilden, Germany) as described in the manufacturer’s instructions with slight modifications. One to four colonies were suspended in 10 mL Mueller–Hinton broth (Oxoid, Hampshire, UK) and left overnight on a shaking incubator set to 250 rpm at 37 °C. Then, the bacterial suspension was centrifuged for 15 min at 4000× *g*. The samples were then incubated again at 37 °C at a 400-rpm shaking incubator for 30–60 min (Innova 4000, New Brunswick Scientific, Hertfordshire, UK). A volume of 100 μL of TE buffer was added to the sample to obtain a final volume of 200 μL in 1.5 mL microcentrifuge tube. Then, 200 μL of the sample was mixed with 400 μL of lysis solution and incubated at 65 °C for 15 min using a heat block (Eppendorf ThermoStat plus, Hamburg, Germany). As per the manufacturer’s instruction, the samples were washed twice using the columns. The DNA pellet was eluted in a final volume of 50–100 μL of nuclease-free H2O (QIAamp^®^ genomic DNA kit, Hilden, Germany). The extracted DNA was aliquoted into 2 vials, which were stored at 4 °C and −80 °C for future use. The DNA quality was evaluated using a 1000 NanoDrop UV spectrophotometer (Thermo Fisher Scientific, Waltham, MA, USA), with an absorbance ratio of 260/280 nm between 1.8 and 2.0, which indicated pure DNA. Gel electrophoresis was performed to ensure there were no RNA or protein contaminants. A GeneRuler 1 kb DNA ladder was used to estimate the DNA fragment sizes.

The extracted DNA was submitted to microbesNG (Birmingham, UK) for whole-genome sequencing (WGS) using Illumina technology. The following steps were performed at the sequencing facility: DNA libraries were prepared with the Nextera XT Library Prep Kit (Illumina, San Diego, CA, USA) following the manufacturer’s instructions, with two modifications: the input DNA was doubled, and the PCR elongation step was extended to 45 s. DNA quantification and library preparation were performed using a Hamilton Microlab STAR automated liquid-handling platform (Hamilton Bonaduz AG, Bonaduz, Switzerland). Pooled libraries were quantified using the Kapa Biosystems Library Quantification Kit (Kapa Biosystems, Wilmington, MA, USA) for Illumina and sequenced on Illumina HiSeq/NovaSeq instruments with a 250 bp paired-end protocol.

Adaptor and quality trimming of raw reads was performed using Trimmomatic v0.30 with a sliding-window threshold of Q15 [75]. Genome assembly was carried out using SPAdes v3.7 [76], and annotation was performed with Prokka v1.11 [77]. The assembled genomes were delivered as contigs, with a minimum coverage depth of 30× to ensure high-quality reads suitable for downstream analyses. The final assemblies and annotations were uploaded for further bioinformatic processing. The sequences were subsequently registered in the MicrobesNG database (https://microbesng.com/ accessed on 16 July 2023). Genome sequences for all *E. coli* and *Klebsiella pneumoniae* isolates were deposited in the GenBank database under accession numbers SAMN47530948–SAMN47530980.

Bioinformatic analyses were performed using tools available through the Center for Genomic Epidemiology (CGE), the Comprehensive Antibiotic Resistance Database (CARD), and the National Center for Biotechnology Information (NCBI). Multi-locus sequence typing (MLST) was conducted via the CGE MLST server (https://www.genomicepidemiology.org, accessed on 4 November 2023) to determine allelic profiles and sequence types (STs) based on six or seven conserved housekeeping genes [78,79,80,81,82]. Whole-genome sequencing (WGS) data were further examined using ResFinder 4.0 and CARD (https://card.mcmaster.ca, accessed on 15 November 2023) to identify antimicrobial resistance genes and associated mechanisms [83,84]. Plasmid profiles of the 26 isolates were characterized using PlasmidFinder 2.1 [85], while virulence-associated genes were identified through VirulenceFinder [86,87,88].

A heat map illustrating the distribution of resistance genes and antimicrobial classes among *E. coli* isolates was generated using Microsoft Excel 2016. Phylogenetic relationships were inferred using the CGE SNP-based phylogeny tool [89,90], and the resulting alignments and tree were visualized in MEGA7 [90,91]. Final annotation and customization of the phylogenetic tree were performed using iTOL (https://itol.embl.de, accessed on 18 November 2023), following the approach described by Letunic and Bork (2007) [92].

### 4.6. Statistical Analysis

For descriptive purposes, categorical variables were presented as numbers and percentages, and continuous variables with mean ± standard deviation, unless otherwise indicated. For inference purposes, categorical variables were compared using the χ^2^ analysis. Antibiotic resistance rates were calculated as percentages. The Fisher test was used to calculate the differences between the isolates’ susceptibility profiles, with a *p*-value of <0.05 considered statistically significant. Sensitivity and specificity analyses of diagnostic tests were performed, and results were visualized using Microsoft Excel. All statistical analyses were performed using IBM Statistical Package for the Social Sciences (SPSS, version 27) software.

## 5. Conclusions

Our findings demonstrate that nitrofurantoin, mecillinam, fosfomycin, temocillin, and nitroxoline retain high in vitro activity against ESBL-producing *E. coli* causing uncomplicated cystitis, thereby meeting the primary objective of identifying effective oral therapeutic alternatives to broad-spectrum agents. From a human health perspective, the use of these agents offers a clinically meaningful strategy to ensure timely and effective treatment of common urinary tract infections while minimizing exposure to cephalosporins and fluoroquinolones, whose overuse is strongly associated with the acceleration of antimicrobial resistance. At the national level, these findings are particularly relevant for Oman, where ESBL-producing Enterobacterales are increasingly reported in both community and healthcare settings. Incorporating these agents into local treatment guidelines and institutional formularies would support evidence-based empirical therapy for simple urinary tract infections, reduce treatment failure, and lower the selective pressure that drives resistance to critically important antimicrobials. Such an approach directly strengthens antimicrobial stewardship programs and contributes to improved patient outcomes, reduced recurrence rates, and preservation of microbiome integrity. Regionally, the results align with resistance trends observed across Asia, where high rates of ESBL production limit oral treatment options for community-acquired infections. The demonstrated efficacy of these narrow-spectrum agents provides a scalable and practical framework for stewardship-driven empirical therapy in similar epidemiological contexts, particularly in resource-constrained settings where access to advanced diagnostics may be limited. Globally, the study supports World Health Organization (WHO) and international antimicrobial stewardship recommendations that advocate for the prioritization of Access-group antibiotics and the de-escalation from Watch and Reserve agents whenever clinically appropriate. By promoting the rational use of effective, lower-tier antimicrobials for uncomplicated infections, these findings contribute to global efforts to curb the emergence and dissemination of multidrug-resistant organisms and to safeguard the long-term effectiveness of last-line therapies. Overall, this study provides clinically actionable evidence that supports sustainable antimicrobial use, reinforces stewardship principles, and has direct implications for public health policy and clinical practice at local, regional, and global levels.

## Figures and Tables

**Table 1:** Prevalence of ESBL- and AmpC β-lactamase-producing *E. coli* across four hospitals.

Hospital	Total *E. coli* Isolates (n)	ESBL-Producing Isolates (n)	ESBL Prevalence (%)	AmpC β-Lactamase-Producing Isolates (n)	AmpC Prevalence (%)
SQUH	200	79	39.5	0	0.0
Khoula Hospital	60	23	38.3	0	0.0
Al-Masarrah Hospital	142	73	51.4	0	0.0
Al-Al-Nahdha Hospital	87	34	39.0	4	4.6

ESBL: Extended Spectrum β-lactamase; AmpC: AmpC β-lactamase.

**Table 2:** Antimicrobial susceptibility profile of *E. coli* (n = 489) and *Klebsiella pneumoniae* (n = 140) among 4 centers; SQUH, Khoula, Al-Masarrah, Al-Al-Nahdha.

	SQUH n (%)		Khoula n (%)		Al Masarah n (%)		Al-Nahdha n (%)	
Antibiotics	*E. coli*	*K. pneumoniae*	*E. coli*	*K. pneumoniae*	*E. coli*	*K. pneumoniae*	*E. coli*	*K. pneumoniae*
Nitrofurantoin	193 (98.5%)	52 (73.5%)	56 (98.2%)	17 (100%)	137 (97.2%)	52 (73.2%)	83 (96.5%)	17 (77.8%)
Cotrimoxazole	142 (71%)	53 (79.2%)	37 (63.8%)	17 (76.5%)	92 (65.7%)	52 (72.5%)	57 (65.5%)	17 (100%)
Ampicillin	60 (30.2%)	42 (0%)	22 (37.9%)	5 (80%)	41 (30.8%)	not tested	28 (32.2%)	not tested
Cefazolin	15 (15.6%)	22 (52.4%)	35 (60.3%)	17 (70.6%)	79 (56%)	52 (68.6%)	49 (56.3%)	not tested
Cefuroxime	109 (56.8%)	53 (80.4%)	31 (57.4%)	17 (70.6%)	39 (41.9%)	52 (47.1%)	45 (56.3%)	17 (94.1%)
Ceftriaxone	123 (61.5%)	51 (82.4%)	34 (59.6%)	17 (76.5%)	84 (61.8%)	52 (76.0%)	52 (59.8%)	17 (94.1%)
Ceftazidime	123 (63.1%)	53 (82.7%)	35 (60.3%)	17 (76.5%)	97 (77.6%)	52 (82.0%)	55 (63.2%)	17 (100%)
Amoxicillin/Clavulanate	88 (79.3%)	40 (91.7%)	49 (94.2%)	17 (93.8%)	89 (78.1%)	52 (79.1%)	67/87 (91.8%)	17 (0%)
Gentamicin	176 (88.4%)	53 (88.7%)	51 (89.5%)	17 (100%)	123 (87.9%)	52 (90.4%)	79 (92.9%)	17 (100%)
Amikacin	198 (99.5%)	52 (96.2%)	56 (100%)	17 (100%)	59 (100%)	15 (93.3%)	85 (98.8%)	not tested
Cefepime	124 (63.6%)	53 (83.0%)	35 (60.3%)	17 (76.5%)	14 (30.4%)	52 (79.2%)	55 (63.2%)	not tested
Piperacillin/Tazobactam	194 (97.5%)	53 (92.2%)	57 (100%)	17 (100%)	59 (98.3%)	15 (50.0%)	85 (100%)	not tested
Ciprofloxacin	82 (62.6%)	44 (82.1%)	34 (64.2%)	17 (93.8%)	75 (58.6%)	52 (61.4%)	55 (63.2%)	not tested
Meropenem	200 (100%)	53 (98.1%)	57 (100%)	17 (100%)	57 (100%)	52 (95.9%)	87 (100%)	17 (100%)
Imipenem	195 (100%)	53 (98.1%)	57 (100%)	17 (100%)	58 (100%)	52 (98.0%)	87 (100%)	17 (100%)
Ceftazidime/Avibactam	140(99.3%)	37 (97.3%)	not tested	not tested	not tested	not tested	not tested	not tested

n: number of susceptible isolates.

**Table 3:** Antimicrobial susceptibility of ESBL- and non-ESBL producing *E. coli* to nitrofurantoin alternatives Mecillinam, Temocillin, Nitroxoline and Fosfomycin.

Name	Non-ESBL Producing *E. coli* (82)			ESBL Producing *E. coli* (30)		
	**S**	**I**	**R**	**S**	**I**	**R**
Mecillinam	100%	0	0	100%	0	0
Temocillin	100%	0	0	27(89.3%)	3 (10.7%)	0
Nitroxoline	100%	0	0	100%	0	0
Fosfomycin	100%	0	0	100%	0	0

**Table 4:** Multi-Locus Sequence Types of *E. coli* (n = 26).

	Isolate(s)	Sequence Type	n
1	EC 8774, EC 8879, EC 2848	ST-131	3
2	EC 2507, EC 4606, EC 6544	ST-174	3
3	EC 6040, EC 9535, EC 3347	ST-1193	3
4	EC 31751, EC 7402, EC 9820	ST-73	3
5	EC 7226, EC 27635	ST-38	2
6	EC 9874, EC 8645	ST-141	2
7	EC 5541, EC 2151	ST-127	2
8	EC 5367	ST-69	1
9	EC 4389	ST-7401	1
10	EC 6067	ST-12	1
11	EC 0159	ST-410	1
12	EC 4990	ST-998	1
13	EC 13668	ST-10	1
14	EC 3063	ST-44	1
15	EC 1286	Novel ST	1

n indicates the number of isolates belonging to each specific sequence type (ST).

**Table 5:** Distribution of efflux resistance genes in *E. coli* n = 26.

Gene	Detected in (Number of Isolates)	Efflux Family	Notes
*marA*	11	Regulator (Global)	Most common regulator
*evgA*	All	RND (Regulator)	Universal in study isolates
*H-NS*	All	H-NS (Nucleoid)	Universal in study isolates
*msbA*	3	ABC	Rare; present in 3 isolates
*mdtN*	3	RND	Rare; present in 3 isolates
*acrB*	2	RND	Found in 2 isolates with AcrE and mdtP
*AcrE*	2	RND	Found in 2 isolates with acrB and mdtP
*mdtP*	2	RND	Found in 2 isolates with acrB and AcrE
*YojI*	1	ABC	Unique gene
*PmrF*	1	LPS modification	Unique gene
*evgS*	1	Two-component	Unique gene
*acrD*	1	RND	Unique gene
*AcrS*	1	RND	Unique gene
*emrA*	1	MFS	Unique gene

**Table 6:** Distribution of resistance genes by ResFinder 4.0 tool, n = 26.

ST	Isolate	Aminoglycoside						Macrolides		Quinolone		Folate Pathway Antagonist					Tetracycline		Beta Lactam						
		*aph(6)-Id*	*aac(6′)-Ib-cr*	*aph(3″)-Ib*	*aac(3)-IIa*	*aadA1*	*aadA5*	*Mrx*	*mph(A)*	*qnrS1*	*gyrA*	*Sul1*	*Sul2*	*dfrA1*	*dfrA14*	*dfrA17*	*tet(A)*	*tet(B)*	AmpC	ESBL					OXA
																			*bla*DHA-*k*	*blaTEM-1B*	*blaSHV-12*	*blaCTX-M-14b*	*blaCTX-M-15*	*blaCTX-M-27*	*blaOXA-1*
**ST-131**	**EC8774**																								
	**EC2848**																								
	**EC8879**																								
**ST-141**	**EC9874**																								
	**EC8645**																								
**ST-174**	**EC2507**																								
	**EC4606**																								
	**EC6544**																								
**ST-1193**	**EC6040**																								
	**EC3347**																								
	**EC9535**																								
**ST-73**	**EC31751**																								
	**EC9820**																								
	**EC7402**																								
**ST-38**	**EC7226**																								
	**EC27635**																								
**ST-127**	**EC2151**																								
	**EC5541**																								
**ST-69**	**EC5367**																								
**ST-7401**	**EC4389**																								
**ST-12**	**EC6067**																								
**ST-410**	**EC0159**																								
**ST-998**	**EC4990**																								
**ST-10**	**EC13668**																								
**ST-44**	**EC3063**																								
**Novel-ST**	**EC1286**																								

Colored cells represent the presence of the corresponding resistance gene in the isolate, while uncolored (blank/white) cells indicate the absence of that gene. Different colors are used only to group resistance genes by antimicrobial class and do not reflect gene abundance or expression.

**Table 7:** Distribution of virulence genes detected in *E. coli*, n = 26.

Virulence Genes	Protein Function	ST-174	ST-1193	ST-73	ST-131	ST-38	ST-141	ST-69	ST-7401	ST-12	ST-410	ST-998	ST-127	ST-10	ST-44	ST- 1258	Total Virulence Genes
		n = 3	n = 3	n = 3	n = 3	n = 2	n = 2	n = 1	n = 1	n = 1	n = 1	n = 1	n = 2	n = 1	n = 1	n = 1	
*cea*	Colicin E1																**2**
*chuA*	Outer membrane hemin receptor																**9**
*clbB*	Hybrid non-ribosomal peptide																**6**
*cnf1*	Cytotoxic necrotizing factor 1																**3**
*colE5*	Colicin E5 lysis protein Lys																**1**
*dhaK*	Dihydroxyacetone kinase (also designated ptnC)																**2**
*eilA*	Salmonella HilA homolog																**1**
*fimH*	Type 1 fimbriae																**13**
*focG*	F1C adhesion																**2**
*fyuA*	Siderophore receptor																**11**
*gad*	Glutamate decarboxylase																**9**
*hlyA*	Hemolysin A																**4**
*hra*	Heat-resistant agglutinin																**6**
*ibeA*	Invasin of brain endothelial cells																**2**
*iha*	Adherence protein																**6**
*ireA*	Siderophore receptor																**3**
*iroN*	Enterobactin siderophore receptor protein																**1**
*irp2*	High molecular weight protein 2 non- ribosomal peptide synthetase																**11**
*iss*	Increased serum survival																**11**
*iucC*	Aerobactin synthetase																**9**
*iutA*	Ferric aerobactin receptor																**8**
*kpsE*	Capsule polysaccharide export inner- membrane protein																**8**
*kpsMII*	Polysialic acid transport protein; Group 2 capsule																**3**
*kpsMII_K1*	Polysialic acid transport protein; Group 2 capsule																**3**
*kpsMII_K5*	Polysialic acid transport protein; Group 2 capsule																**1**
*lpfA*	Long polar fimbriae																**3**
*mchB*	Microcin H47 part of colicin H																**2**
*mchC*	MchC protein																**1**
*mchF*	ABC transporter protein MchF																**1**
*mcmA*	Microcin M part of colicin H																**2**
*neuC*	Polysialic acid capsule biosynthesis protein																**3**
*nlpI*	lipoprotein NlpI precursor																**2**
*ompT*	Outer membrane protease (protein protease 7)																**8**
*papA_F11*	Major pilin subunit F11																**1**
*papA_F43*	Major pilin subunit F43																**3**
*papA_F48*	Major pilin subunit F48																**1**
*papA_F7-2*	Major pilin subunit F7-2																**1**
*papA_F9*	Major pilin subunit F9																**1**
*papA_fsiA_F16*	Major pilin subunit F16																**1**
*papC*	Outer membrane usher P fimbriae																**2**
*pic*	serine protease autotransporters of Enterobacteriaceae (SPATE)																**1**
*sat*	serine protease autotransporters of Enterobacteriaceae (SPATE)																**1**
*senB*	Plasmid-encoded enterotoxin																**8**
*sfaD*	S fimbrial/F1C minor subunit																**1**
*sfaS*	S-fimbriae minor subunit																**1**
*sitA*	Iron transport protein																**11**
*tcpC*	Tir domain-containing protein																**6**
*terC*	Tellurium ion resistance protein																**5**
*traT*	Outer membrane protein complement resistance																**8**
*usp*	Uropathogenic specific protein																**4**
*vat*	serine protease autotransporters of Enterobacteriaceae																**3**
*yehA*	Outer membrane lipoprotein, YHD fimbrial cluster																**3**
*yehB*	Usher, YHD fimbrial cluster																**1**
*yehC*	Chaperone, YHD fimbrial cluster																**1**
*yehD*	Major pilin subunit, YHD fimbrial cluster																**1**
*yfcV*	Fimbrial protein																**5**

Color indicates the presence and distribution of virulence genes among sequence types (STs). Shaded cells represent the number of isolates within each ST carrying the indicated virulence gene, based on the total *n* for that ST. Unshaded cells indicate absence.

## Data Availability

All supporting data can be found in the manuscript. All whole-genome sequencing data are deposited in DDBJ/ENA/GenBank under the accessions SAMN47530948–SAMN47530980. The version described in this paper is version SAMN47530948–SAMN47530980. The raw data supporting the conclusions of this article will be made available by the authors without undue reservation.

## Abbreviations

The following abbreviations are used in this manuscript:

ABC	ATP-binding cassette
AmpC	Ambler class C cephalosporinases
AMR	Antimicrobial Resistance
AMS	Antimicrobial stewardship
ATCC	American Type Culture Collection
AUC	Acute uncomplicated cystitis
BL	Beta-lactamase
BLI	Beta-lactamase inhibitor
CARD	Comprehensive Antibiotic Resistance Database
caUTI	Community-acquired urinary tract infection
CEC	Cefaclor
CFM	Cefixime
CGE	Genomic Epidemiology
CLSI	Clinical and Laboratory Standards Institute
CRO	Ceftriaxone
CXM	Cefuroxime
DDST	Double-Disk Synergy Test
*E. coli*	*Escherichia coli*
EB	Elution buffer
EDs	Emergency departments
ESBL	Extended-spectrum beta-lactamases
FAMCO	Family Medicine and Public Health
FOS	Fosfomycin
FQ	Fluoroquinolones
GBS	Group B Streptococcus
GC	Guanine-cytosine
ICU	Intensive care unit
KAP	Knowledge, Awareness and Practice
KPC	*Klebsiella pneumoniae* carbapenemase
KZ	Cefazolin
LB	Luria Broth
MDR	Multidrug resistant
MDRO	Multidrug resistant organisms
MEC	Mecillinam
MIC	Minimum inhibitory concentration
MLST	Multilocus sequence typing
MOH	Ministry of health
MPH	Macrolide phosphotransferase
MREC	Medical Research Ethics Committee
MRN	Medical records number
MRSA	Methicillin-resistant *Staphylococcus aureus*
MSU	Midstream Specimen of Urine
NCBI	National Center for Biotechnology Information
NFT	Nitrofurantoin
NI	Nitroxoline
NS	Non-susceptible
OPD	Outpatient departments
PDR	Pan-drug resistance
PRO	patient-reported outcome
RND	resistance-nodulation-cell division
SNPs	Single Nucleotide Polymorphisms
SQUH	Sultan Qaboos University Hospital
TMO	Temocillin
TMP-SMX	Trimethoprim-sulfamethoxazole
TPB	Theory Planned Behavior
UPEC	Uropathogenic *E. coli*
UTI	Urinary tract infection
WGS	Whole Genome Sequencing
WHO	World Health Organization
XDR	Extensive drug resistance
